# Supplementary material for: Patterns of Intron Gain and Loss in Fungi
Source: PLoS Biol. 2004 Nov 30;2(12):e422. doi: 10.1371/journal.pbio.0020422 (PMC532390; doi:10.1371/journal.pbio.0020422)
Supplement: Table S1 — Also available at http://genes.mit.edu/NielsenEtAl/. (4.3 MB ZIP). [file pbio.0020422.st001.zip › NielsenEtAl/html/292.html]

AN7052.1.NCU06596.1.MG01705.1.FG05260.1


```
 CLUSTAL W (1.82) Multiple Sequence Alignments - Introns Inserted


Sequence 1: NCU06596.1	334 aa
Sequence 2: MG01705.1	314 aa
Sequence 3: FG05260.1	301 aa
Sequence 4: AN7052.1	321 aa
Alignment Length: 377 aa
Number Identitical Residues: 98 aa
Alignment Score (without introns) 5411


MG01705.1 	MNG------------NTGTGRKQAAIVAATAAAAVVSSPAAFSKPD-------MPTSSSK
NCU06596.1	MKEGTPAPVSAASPLDTAAGLKTSHSHLAGLATQTQKTRAALLGADGGDVVDTKPTAMTS
FG05260.1 	--------------------MSKSNIMKDSDAPALALSGATT-------------TEQIT
AN7052.1  	-------------------------MPQALQDAAFIPS----------------------
          	                                .    :                      

MG01705.1 	SQRVWNTKNLGWRLAADAISAAAAATMVAPLISIIDR2SIMENASGRRSLIESVKSSLAN
NCU06596.1	TTSKWNTKNLGLRLGADLVSAAAAAVLVAPVISIIDR2SIMENASGAAPLLTSLRRSLRS
FG05260.1 	TKNTWNTKNLAPRLGADFLSAASAASMVAPLIAIIDR2SIMENASGANTLINSVKSSLRT
AN7052.1  	PTKSWNTNRLGARFGVDIVSAATAGALTCPVITVIDR2AIIEKAAKGVPIRETITSAFRS
          	.   ***:.*. *:..* :***:*. :..*:*::*** :*:*:*:   .:  ::  :: .

MG01705.1 	LIMRPHTIIFARPFALICMTYGGTYLTANTLDTATSTVKNKP-ATLVTSGTAKFVASSTA
NCU06596.1	LLSSPRSTILSRPFGLIFLLYGGTYLTANTLDTTVATVQNNPNPAHVTSGSSKFFASSAA
FG05260.1 	LITRPHTLLFSKPTALIFCLYGGTYLTANAVDTSFSTVNNKP-ASSVTAGTTKFAASSAA
AN7052.1  	IIRKPSGFFLGTPFLLIYTLYTSTYLTANTIDTVTSMLKDKP-FSTVFPGTAKFITTTAV
          	::  *   ::. *  **   * .******::**  : ::::*  : * .*::** ::::.

MG01705.1 	NTGLGIYKDQVFARMFG----PPGATPRP-------------------------------
NCU06596.1	NIGLCIYKDQVFVRLFG----PPGATPRP-------------------------------
FG05260.1 	NIGVCIYKDQVFVRMFG----PPGVVPRP-------------------------------
AN7052.1  	NMGICVYKDARFARIFGKQSSQPQGQPQPQPPLAKSPSNGLFTATMPASCHPSPLTVGAP
          	* *: :***  *.*:**..:: *   *:*... :.:.:..  ::: .::. .:. : .:.

MG01705.1 	-VPLPSYLLFAMRDAVTIFASFNLPIMLAPYLNNHLSDGFKKHISGQTLAQFAAPAAIQI
NCU06596.1	-VGLPSYLLFAARDCMTIFASFNVPPLLGPVLTERLSKDAQKWVSGQTMAQFAAPAVVQL
FG05260.1 	-VPLASYGLFALRDCMTIFASFNVPPLLGPILDEKFSAEMKKRVSGLYAAQFMAPAMVQF
AN7052.1  	KVPKISYTLFCLRDSITIFASFNIPALIAPSIPDSIAATPAMKAA---IAQFSCPALMQF
          	.*   ** **. **.:*******:* ::.* : : ::       :    *** .** :*:

MG01705.1 	ISTPPHLLGLDLYNR-PTCASS-GSPTWAERWASVRSNWAISSCARVCRIVPAFGIGGVV
NCU06596.1	FSTPVHLLGLDMYNR-PSGGSG-AAPSWGERWSLVKKNWAVSVAARICRIVPAFGVGGVV
FG05260.1 	ISTPMHLFGLDLYNR-QATGPGGAVVSLRDRLDLVRRNWFISSIARICRIVPAFGVGGVV
AN7052.1  	VSTPMHLLGLDLYNRQPPGGLG-----WRERVGRIRRDYVASCFARMGKIVPAYGIGGVV
          	.*** **:***:***. . . .       :*   :: ::  *  **: :****:*:****

MG01705.1 	NFKVRRQFMEKLT-----
NCU06596.1	NMKVRKGLMERLS-----
FG05260.1 	NMKVRKNLMTKLE-----
AN7052.1  	NVRMRAELMGWLEGLDQA
          	*.::*  :*  * . ..:
```
